# Effects of human adipose tissue- and bone marrow-derived mesenchymal stem cells on airway inflammation and remodeling in a murine model of chronic asthma

**DOI:** 10.1038/s41598-022-16165-8

**Published:** 2022-07-14

**Authors:** Joon Young Choi, Jung Hur, Sora Jeon, Chan Kwon Jung, Chin Kook Rhee

**Affiliations:** 1 Division of Pulmonary and Critical Care Medicine, Department of Internal Medicine, Incheon St. Mary’s Hospital, College of Medicine, The Catholic University of Korea, Seoul, Republic of Korea; 2 Division of Pulmonary and Critical Care Medicine, Department of Internal Medicine, Seoul St. Mary’s Hospital, College of Medicine, The Catholic University of Korea, 222 Banpodaero, Seochogu, Seoul, 06591 Republic of Korea; 3 Cancer Research Institute, College of Medicine, The Catholic University of Korea, Seoul, Republic of Korea; 4 Department of Hospital Pathology, Seoul St. Mary’s Hospital, College of Medicine, The Catholic University of Korea, 222 Banpodaero, Seochogu, Seoul, 06591 Republic of Korea

**Keywords:** Respiratory tract diseases, Mesenchymal stem cells

## Abstract

It is challenging to overcome difficult-to-treat asthma, and cell-based therapies are attracting increasing interest. We assessed the effects of mesenchymal stem cell (MSC) treatments using a murine model of chronic ovalbumin (OVA)-challenged asthma. We developed a murine model of chronic allergic asthma using OVA sensitization and challenge. Human adipose-derived MSCs (hADSCs) or human bone marrow-derived MSCs (hBMSCs) were administered. We measured the levels of resistin-like molecule-β (RELM-β). We also measured RELM-β in asthma patients and normal controls. OVA-challenged mice exhibited increased airway hyper-responsiveness, inflammation, and remodeling. hBMSC treatment remarkably decreased airway hyper-responsiveness but hADSC treatment did not. Both MSCs alleviated airway inflammation, but hBMSCs tended to have a more significant effect. hBMSC treatment reduced Th2-cytokine levels but hADSC treatment did not. Both treatments reduced airway remodeling. The RELM-β level decreased in the OVA-challenged control group, but increased in both treatment groups. We found that the serum level of RELM-β was lower in asthma patients than controls. MSC treatments alleviated the airway inflammation, hyper-responsiveness, and remodeling associated with chronic asthma. hBMSCs were more effective than hADSCs. The RELM-β levels increased in both treatment groups; the RELM-β level may serve as a biomarker of MSC treatment efficacy.

## Introduction

Asthma is a chronic inflammatory disease characterized by airway inflammation, airway hyperresponsiveness (AHR), and remodeling^1^. Asthma affects 340 million people worldwide; the annual number of cases has been increasing for decades^2^. The current mainstays of treatment are corticosteroids and long-acting bronchodilators, but recent advances in biologics may greatly aid the treatment of severe asthma^3^; such patients currently have unmet needs^4^. About 4–5% of all asthma patients have severe asthma^5,6^, which accounts for over 60% of the asthma-related economic burden^7^. New drugs are urgently needed^1,8^.

Mesenchymal stem cells (MSCs) are pluripotent, undifferentiated progenitor cells present in various organs; they play roles in tissue regeneration and immunomodulation^9,10^. MSC treatments have shown promise in patients with hematological diseases, graft-versus-host disease, diabetes, multiple sclerosis, Crohn’s disease, ulcerative colitis, and lupus, and in patients undergoing kidney transplantation^11^. Recent studies on animal models of asthma have reported encouraging outcomes of MSC treatments; asthmatic features were alleviated^12–15^. MSC treatments target the immune system, affecting inflammatory cells including T-lymphocytes, dendritic cells (DCs), macrophages, and epithelial cells^11,16,17^. One of the most important immunological mechanisms of allergic asthma is Th1/Th2 cell ratio imbalance, which was restored by MSC treatments^18–20^. In one case series (three patients with severe asthma), administration of autologous bone-marrow mononuclear cells (BMMCs) safely enhanced patient quality of life^21^. Although the number of patients was small and the placebo-controlled randomized design was not used, the results were encouraging.

We previously described the beneficial effects of human bone marrow mesenchymal stem cells (hBMSCs) and human adipose-derived stem cells (hADSCs) in a murine model of acute asthma^22^. However, the acute ovalbumin (OVA)-challenged asthma model has a limitation, in that airway remodeling (an important feature of human asthma pathophysiology)^23^ is lacking.

Resistin-like molecule-β (RELM-β) is a small, secreted cysteine-rich protein of the RELM family that includes RELM-α, RELM-γ, and resistin^24,25^. The RELM family is associated with various infectious, metabolic, inflammatory, and malignant diseases; recent work focused on its role in cardiothoracic diseases^24–26^. The data from animal models of asthma are equivocal; RELM-β has been reported to play both pro-inflammatory^27,28^ and anti-inflammatory^29^ roles. A few reports described associations between RELM-α levels and MSC recruitment/proliferation. However, to the best of our knowledge, no study has investigated the role of RELM-β in MSC treatments, especially of asthma. Here, we aimed to evaluate the effects of MSC treatments using a murine model of chronic OVA-challenged asthma. We evaluated the effects of MSC on airway inflammation, airway hyperresponsiveness, and remodeling. We aimed to compared the effects of hBMSCs and hADSCs, and measured RELM-β levels in mice and humans to explore its role in asthma pathophysiology and MSC treatments.

## Results

### Effect of hMSCs on AHR

**Figure 1:**
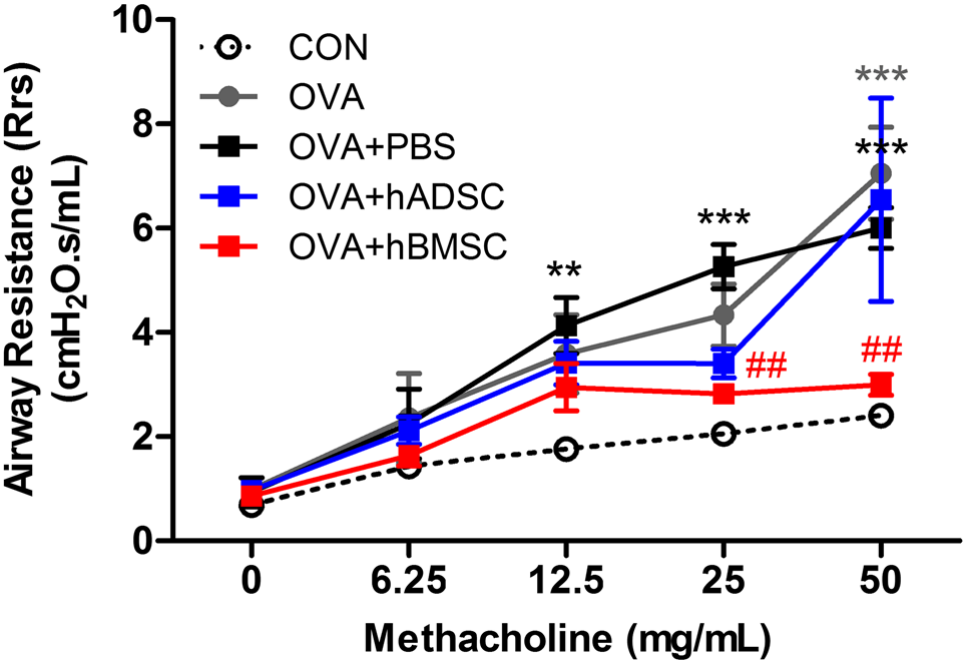
Effect of hADSC and hBMSC on AHR to Mch. AHR, airway hyperresponsiveness. The OVA + hBMSC group presented remarkable AHR decreases at Mch doses of 25 and 50 mg/mL, but no significant decrease was shown in the OVA + hADSC group. *OVA* ovalbumin, *PBS* phosphate buffer saline, *hADSC* human adipose-derived stem cell, *hBMSC* human bone marrow mesenchymal stem cell. **P < 0.01, ***P < 0.001 compared to control, ^##^*P* < 0.01 compared to the OVA + PBS group.

The OVA + PBS group exhibited more airway resistance than the CON group at Mch doses of 12.5, 25, and 50 mg/mL (Fig. 1). The OVA + hBMSC group evidenced remarkable AHR decreases at Mch doses of 25 and 50 mg/mL, but no significant decrease was evident in the OVA + hADSC group.

### Effect of hMSCs on airway inflammation

**Figure 2:**
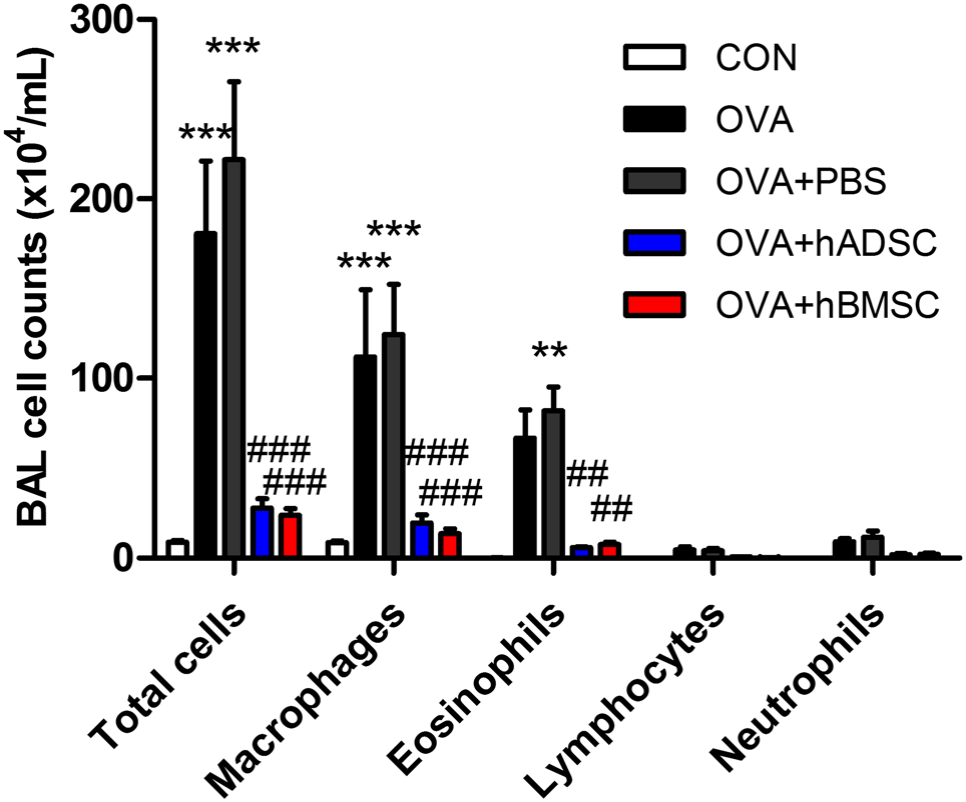
Effect of hADSC and hBMSC on total/differential cell counts in BAL fluid. The OVA group evidenced significant increases in the numbers of total cells, macrophages, and eosinophils in BAL fluid compared to the hADSC- and hBMSC-treated groups. *BAL* bronchoalveolar lavage, *OVA* ovalbumin, *PBS* phosphate buffer saline, *hADSC* human adipose-derived stem cell, *hBMSC* human bone marrow mesenchymal stem cell. **P < 0.01, ***P < 0.001 compared to control, ^##^*P* < 0.01, ^###^*P* < 0.001 compared to the OVA + PBS group.

**Figure 3:**
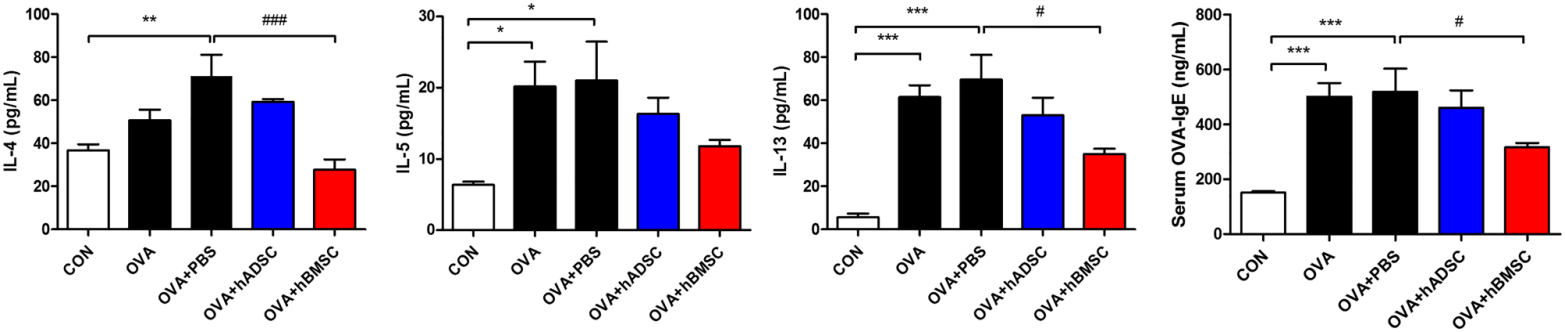
Effect of hADSC and hBMSC on levels of Th2 cytokines in BAL fluid. Repeated OVA challenge induced significant increases in IL-5 and IL-13. The OVA + PBS group mice showed significant increases in IL-4, IL-5, and IL-13 levels; hBMSC treatment significantly attenuated the increases of IL-4 and IL-13. The OVA + hADSC group mice did not show a significantly change in Th2 cytokine levels. *BAL* bronchoalveolar lavage, *OVA* ovalbumin, *PBS* phosphate buffer saline, *hADSC* human adipose-derived stem cell, *hBMSC* human bone marrow mesenchymal stem cell. *P < 0.05, **P < 0.01, ***P < 0.001 compared to control, ^#^*P* < 0.05, ^###^*P* < 0.001 compared to the OVA + PBS group.

**Figure 4:**
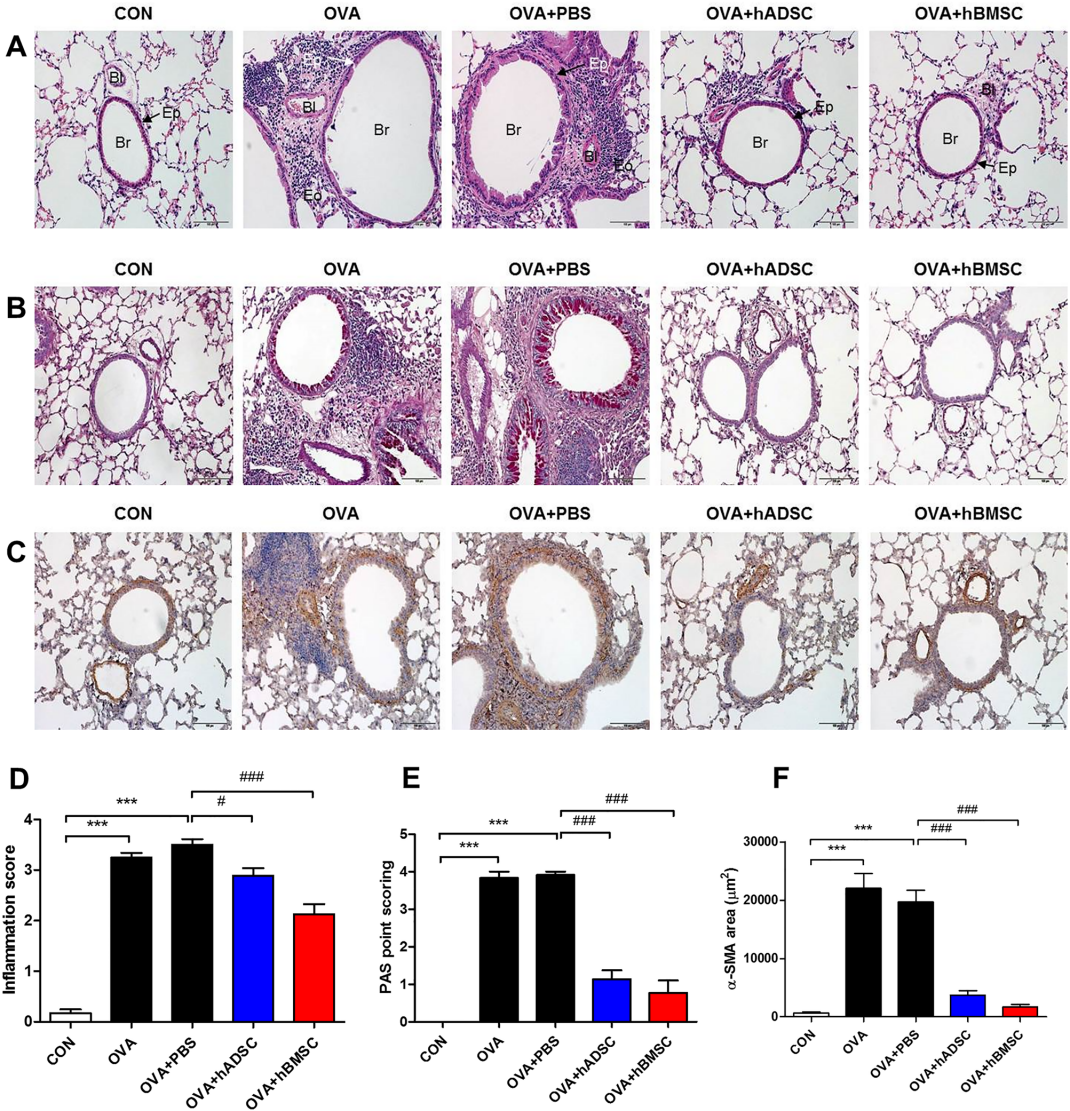
Effect of hADSC and hBMSC on (**A**) lung pathology (H&E stain) and (**D**) inflammation score. H&E staining exhibited significant infiltration of inflammatory cells in the subepithelial, peribronchial, and perivascular lesions of the OVA and OVA + PBS groups compared to the CON group. Compared to the OVA + PBS group, both the OVA + hADSC and OVA + hBMSC groups revealed attenuation of inflammatory cell infiltration, especially the latter group. The inflammatory scores showed significantly attenuated airway inflammation, in both hMSC-treated group but the effect tended to be better in the OVA + hBMSC than OVA + hADSC group. Effect of hADSC and hBMSC on goblet cell hyperplasia in lung tissues, demonstrated by (**B**) PAS staining and (**E**) PAS score. PAS-stained sections showed prominent goblet cell hyperplasia in the OVA and OVA + PBS groups; both MSC treatments attenuated this. The PAS scores were significantly higher in the OVA and OVA + PBS groups than the MSC treatment groups. Effect of hADSC and hBMSC on the area of peribronchial airway smooth muscle. (**C**) Peribronchial α-SMA was immunostained in lung section. (**F**) The immunostained area was quantified by using light microscope. Repeat OVA challenge significantly increased the immunostained area of peribronchial α-SMA compared to that of the CON group; both MSC treatments alleviated this. *OVA* ovalbumin, *PBS* phosphate buffer saline, *hADSC* human adipose-derived stem cell, *hBMSC* human bone marrow mesenchymal stem cell, *Br* bronchus, *Bl* blood vessel, *Ep* epithelium, *Eo* eosinophil. ***P < 0.001 compared to control, ^#^*P* < 0.05, ^###^*P* < 0.001 compared to the OVA + PBS group.

The OVA group exhibited significant increases in the numbers of total cells, macrophages, and eosinophils in BAL fluid compared to the hADSC- and hBMSC-treated groups (Fig. 2). Although there were trends to show decreased lymphocytes and neutrophils in MSC-treated groups, there were no statistical significance. In terms of BAL fluid Th2 cytokines, repeated OVA challenge induced significant increases in IL-5 and IL-13. The OVA + PBS group mice showed significant increases in IL-4, IL-5, and IL-13 levels; hBMSC treatment significantly attenuated the increases of IL-4 and IL-13 (Fig. 3). The OVA + hADSC group mice did not show a significantly change in Th2 cytokine levels. Serum OVA-specific immunoglobulin (Ig)-E was measured by ELISA, and showed that OVA and OVA + PBS group significantly increased OVA-specific IgE levels and hBMSC treatment group decreased compared with OVA + PBS group. However, OVA + hADSC group did not show significant change. Histopathological (H&E) staining revealed significant infiltration of inflammatory cells (including eosinophils) in the subepithelial, peribronchial, and perivascular lesions of the OVA and OVA + PBS groups compared to the CON group (Fig. 4A). Compared to the OVA + PBS group, both the OVA + hADSC and OVA + hBMSC groups exhibited attenuation of inflammatory cell infiltration, especially the latter group. The inflammatory scores were in line with these observations; both MSC treatments significantly attenuated airway inflammation, but the effect tended to be better in the OVA + hBMSC than OVA + hADSC group (Fig. 4D).

### Effects of hMSCs on airway remodeling

**Figure 5:**
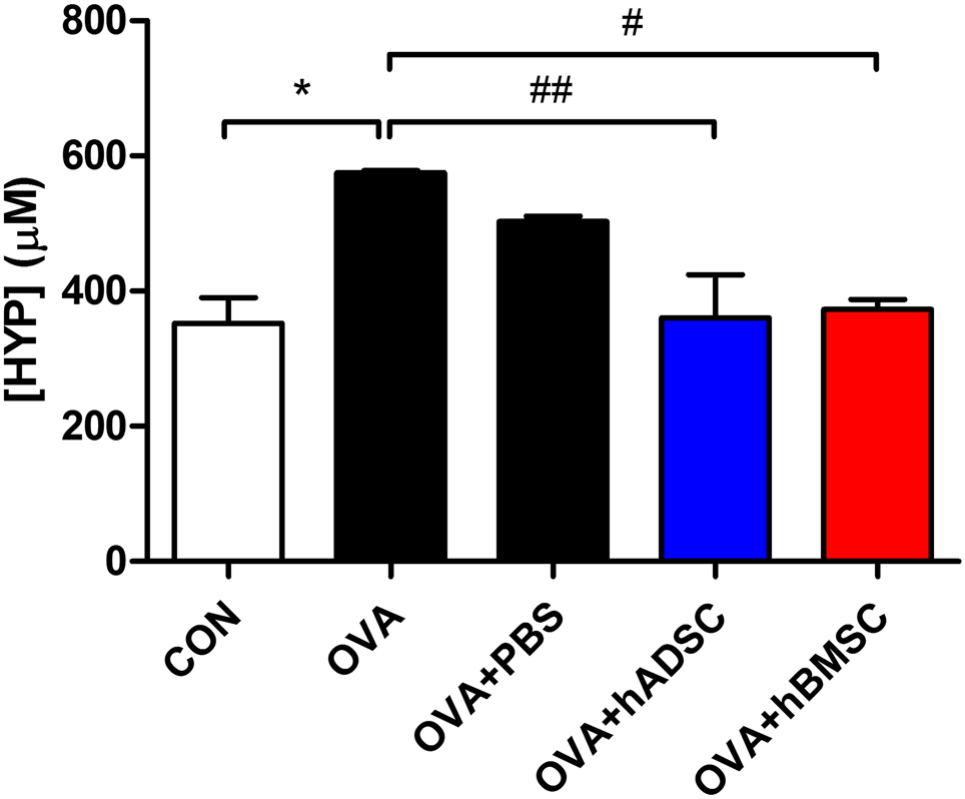
Effect of hADSC and hBMSC on total collagen levels measured by hydroxyproline assay. Lung collagen deposition was increased by repeated OVA challenge, but this was suppressed by both MSC treatments. *OVA* ovalbumin, *PBS* phosphate buffer saline, *hADSC* human adipose-derived stem cell, *hBMSC* human bone marrow mesenchymal stem cell. *P < 0.05 compared to control, ^#^*P* < 0.05, ^##^*P* < 0.01 compared to the OVA + PBS group.

PAS-stained sections exhibited prominent goblet cell hyperplasia in the OVA and OVA + PBS groups; both MSC treatments attenuated this (Fig. 4B). The PAS scores were significantly higher in the OVA and OVA + PBS groups than the MSC treatment groups (Fig. 4E). Repeat OVA challenge significantly increased the immunostained area of peribronchial α-SMA compared to that of the CON group; both MSC treatments alleviated this (Fig. 4C,F). Lung collagen deposition (assessed by hydroxyproline assay) was increased by repeated OVA challenge, but this was suppressed by both MSC treatments (Fig. 5).

### RELM-β levels in mice with chronic asthma

**Figure 6:**
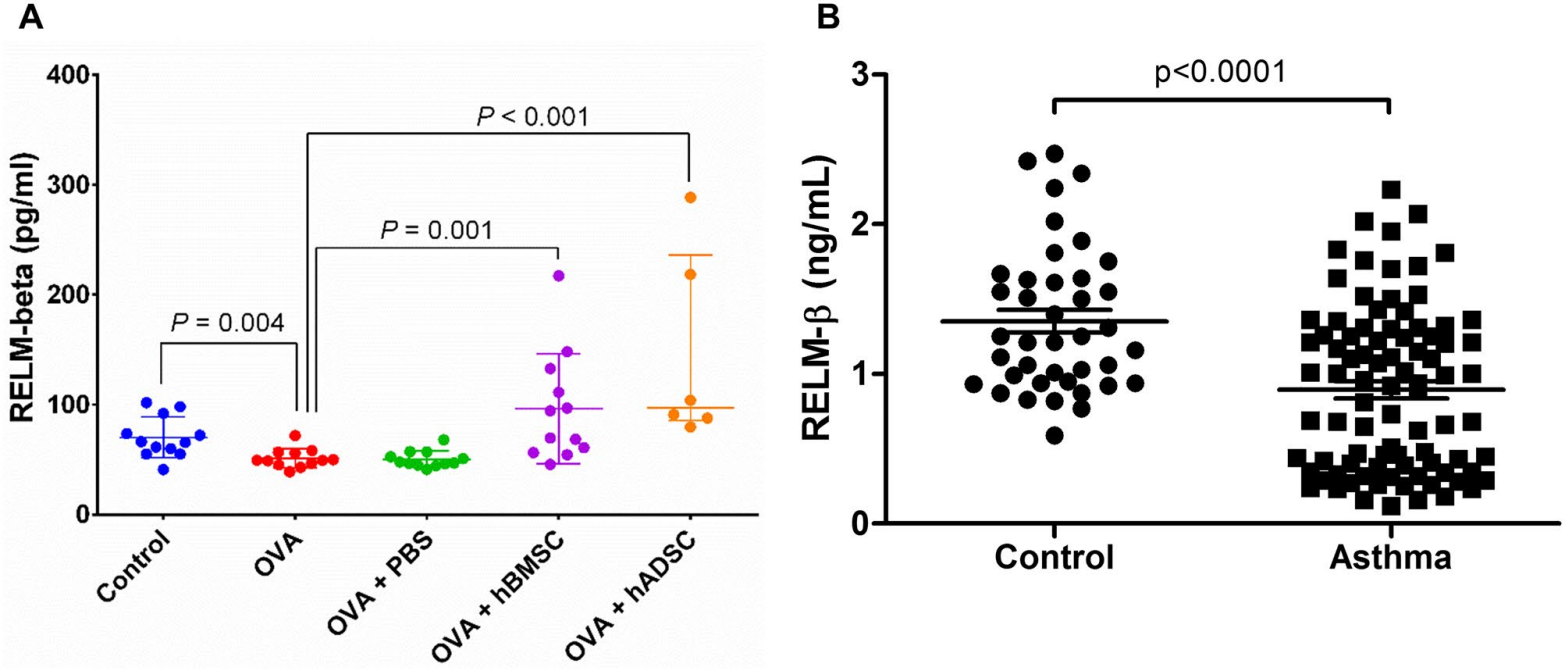
(**A**) Difference of RELM- β levels in (**A**) BAL fluid of hADSC and hBMSC treated mice compared to control, and serum RELM- β levels (**B**) between asthma patients and normal control. Repeated OVA challenge significantly decreased the RELM-β level, while both MSC treatments significantly increased the RELM-β level. Also, the serum RELM-β level was significantly lower in asthma patients compared to controls. *OVA* ovalbumin, *PBS* phosphate buffer saline, *hADSC* human adipose-derived stem cell, *hBMSC* human bone marrow mesenchymal stem cell, *RELM-β* resistin-like molecule-β.

Repeated OVA challenge significantly decreased the RELM-β level, while both MSC treatments significantly increased the RELM-β level (Fig. 6A).

### RELM-β levels in asthma patients

General characteristics of asthma patients were as follows: median age were 66 [59.0–73.0]; 92.9% were male; BMI were 24.3 [21.1–26.2]; FVC were 3.4 [2.8–4.1] L; FEV1 were 1.8 [1.1–2.2] L, FEV1/FVC were 49.5 [37.0–58.0] %; BDR were 15.0 [7.0–23.0] %; peripheral eosinophil cell count were 144.4 [82.8–486.0] and fractional exhaled nitric oxide (FeNO) were 25.0 [13.0–37.0] ppb. In normal control group, median age were 55.5 [50.0–65.0]; all subjects were male and BMI were 23.7 [22.3–26.1]. The serum RELM-β level was significantly lower in asthma patients compared to controls (Fig. 6B).

## Discussion

We found that hMSCs alleviated the OVA-challenged chronic asthma features of mice, i.e., AHR, inflammation, and remodeling. hBMSCs were more effective than hADSCs, and only hBMSCs significantly attenuated AHR. The RELM-β levels were reduced in the BAL fluid of OVA-challenged mice and the sera of asthmatic patients, but increased in OVA-challenged nice that received MSCs. This study is the first to report that the RELM-β level may affect the outcome of MSC treatment; the RELM-β level may serve as a biomarker of treatment efficacy.

We previously showed that hMSCs alleviated AHR and airway inflammation in a mouse model of acute OVA-challenged asthma^22^. hMSC dosage and timing greatly affect the asthmatic features. Here, we used a 3-month chronic murine asthma mouse model, because this reflects human asthma better than the acute model due to consideration of airway remodeling^23,30–35^.

Previous studies reported that MSCs were effective for treating chronic asthma. Firinci et al. showed that BMSC treatment of mice with chronic OVA-challenged asthma alleviated the airway remodeling characterized by goblet cell hyperplasia, and the increased thickness of the basement membrane/subepithelial smooth muscle layer^36^. Ge et al., using an OVA-challenged chronic asthma model, reported favorable airway inflammation and remodeling outcomes in a BMSC treatment group. They used enhanced green fluorescent protein (EGFP)-expressing BMSCs and immunofluorescence staining to determine whether BMSCs participated in lung regeneration. EGFP-BMSC levels were extremely low in the lungs 50 days after transplantation, and immunofluorescent staining indicated that CK, a-SMA, and SPC were absent, suggesting that BMSCs may not participate in lung regeneration but could exert immunomodulatory and anti-inflammatory effects. Dalouchi et al. showed that human amniotic membrane MSC-conditioned medium alleviated airway inflammation and fibrosis in an OVA-challenged asthma model, again suggesting immunomodulatory effects (rather than active participation in lung regeneration)^37^.

ADSCs, compact bone-derived MSCs, human placenta-derived MSCs, induced pluripotent stem cells (iPSCs) and mesenchymoangioblast-derived MSCs also improved asthmatic features in an allergic asthma model^14,38–42^. Abreu et al. reported that BMSCs provided better outcomes than ADSCs and lung tissue-derived MSCs in terms of lung mechanics, airway inflammation, and remodeling, in agreement with our findings^43^. The mechanisms remain obscure, but the different soluble factors and microenvironments among MSCs may play a role. Abreu et al. found that BMSCs accelerated macrophage M2 polarization, which is important in terms of both wound repair and anti-inflammation^43^. Therapeutic effects of MSCs on non-allergic^44–46^ and occupational asthma ^20,47,48^ have also been reported.

The immunomodulatory actions of MSCs in asthma models involve effects on various inflammatory cells including T cells, DCs, macrophages, and epithelial cells^16^. The main immunological component of allergic asthma pathogenesis is a Th1/Th2 ratio imbalance (Th2 dominance)^19^. The levels of Th2-associated cytokines (IL-4, IL-5, and IL-13) increased in various allergic asthma models; MSC treatments generally restored the Th1/Th2 balance and reduced the cytokine levels, as we also observed^12,18,20,42,43^. MSCs hindered the maturation and migration of lung DCs to lymph nodes, thereby alleviating Th2 inflammation. MSCs inhibited DC activation of naïve and effector Th2 cells, and suppressed the production of chemokines that attract Th2 cells to the airway.

The mammalian RELM family of secreted proteins are characterized by conserved, cysteine-rich carboxyl domains^29^. RELMs play important roles in various inflammatory/fibrotic diseases, malignancies, and metabolic diseases, as well as in immunity to microbial infection^26^. Of the various RELM proteins, RELM-β and resistin are produced by humans^24^. RELM-β has been associated with both a pro-inflammatory or anti-inflammatory asthmatic course. Mishra et al., using an allergic asthma model, reported elevated RELM-β mRNA expression in asthmatic lungs, which was attenuated by blockade of IL-4Rα (a subunit shared by the IL-4 and IL-13 receptors)^28^. Intratracheal administration of recombinant murine RELM-β exacerbated airway inflammation and remodeling in naïve mice, but attenuated remodeling in genetically deficient RELM-β mice. Fang et al. found that RELM-β deposits in the extracellular matrix of human asthma patients exacerbated airway remodeling by stimulating lung fibroblast proliferation and myofibroblast differentiation^27^. However, LeMessurier et al. reported the opposite results. They compared *Retnlb* wild-type and knockout mice sensitized with *Aspergillus fumigatus* antigens (a model of severe asthma)^29^. RELM-β absence exacerbated airway inflammation, hyperresponsiveness, and remodeling, as we also found. The reason for the equivocal findings is unclear, although studies reporting negative effects of RELM-β had shorter-term antigen exposure times than studies describing protective effects^27–29^. Acute and chronic exposure to allergens may affect asthma progression differently.

We found that the RELM-β serum level was lower in asthma patients than controls. In contrast, Fang et al. reported elevated RELM-β levels in the BAL fluid and bronchial mucosa of asthma patients compared to controls^49^. They also reported that increased numbers of bronchial mucosa RELM-β-positive cells were associated with unfavorable outcomes, negatively correlated with the forced expiratory volume in 1 s, and positively correlated with the number of MUC5AC-positive cells^49^. However, both studies included small numbers of patients (84 and 40, respectively); further studies with more subjects are needed.

In conclusion, we found that MSC treatment alleviated the airway inflammation, hyperresponsiveness, and remodeling seen in chronic asthma. The effects of hBMSCs were more profound than those of hADSCs. MSCs increased the RELM-β levels, where RELM-β may serve as a biomarker of MSC treatment outcomes.

## Methods

### Animals and experimental design

Six-week-old female BALB/c mice (Orient Bio, Gyeonggi-do, Korea) were used in all experiments. The mice were randomly allocated to the following groups: control (CON, n = 6); OVA challenge (OVA, n = 6); OVA challenge + phosphate-buffered saline (PBS) as vehicle (OVA + PBS, n = 8); OVA challenge + hADSCs (OVA + hADSC, n = 8); and OVA challenge + hBMSCs (OVA + hBMSC, n = 8). All animal procedures were performed in accordance with the ARRIVE guideline, the Laboratory Animal Welfare Act, Guide for the Care and Use of Laboratory Animals, and Guidelines and Policies for Rodent Experiments of the Institutional Animal Care and Use Committee (IACUC), School of Medicine, Catholic University of Korea (approval no. CUMC-2020–0144-03).

### Sensitization and antigen challenge

OVA sensitization and challenge were performed as described previously^23^. Mice were immunized via subcutaneous injection of 25 μg OVA (chicken egg albumin, grade V; Sigma-Aldrich, St. Louis, MO, USA) absorbed to 1 mg of aluminum hydroxide (Sigma-Aldrich, Milwaukee, WI, USA) in 200 μL of PBS. Injections were administered on days 0, 7, 14, and 21, followed by intranasal OVA (20 μg/50 μL in PBS) challenge on days 33, 35, and 37. The OVA challenge was repeated twice weekly for 3 months after the mice had been anaesthetized with isoflurane (Vedco, St. Joseph, MO, USA). Age- and sex-matched control mice received PBS without OVA. Mice were sacrificed 24 h after the last intranasal OVA challenge, and bronchoalveolar lavage (BAL) fluid and lung tissues were collected.

### Administration of hADSCs and hBMSCs

hADSCs were provided by ACB cell bank, plastic surgery of catholic university, Seoul Mary’s Hospital of Korea, Seoul, Korea. hBMSCs were provided by Catholic Institute of Cell Therapy. Both MSCs were obtained from 17 adult donors after approval was granted by the Institutional Review Board of our center, as described previously^22,50^. Donor information of hADSCs were as follow: mean age was 44.8 ± 13.6, 3 patients (17.6%) were male, mean BMI was 25.1 ± 5.4. Sample tissue, diagnosis and operation name are presented in the supplementary material. The donor information of hBMSC was not disclosed.

Vials of frozen hADSCs and hBMSCs were thawed and expanded according to the instructions of the supplier. All hMSCs were passage 3–4. In total, 2.5 × 10^7^ /kg hADSCs or hBMSCs in 100 μL PBS were injected (via an insulin syringe) into mouse tail veins 5 days prior to sacrifice.

### AHR measurement

The methacholine (Mch) (Sigma-Aldrich) AHR was measured 24 h after the last OVA challenge using the flexiVent system (SCIREQ, Montreal, QC, Canada), as described previously^23^, with the mice under anesthesia induced via intraperitoneal injection of a 1:4 (w/w) mixture of rompun and Zoletil. The tracheas were exposed, cannulated, and connected to animal ventilators (tidal volume = 10 mL/kg at a rate of 150 breaths/min, with a positive end-expiratory pressure of 2 cm H_2_O [normal mouse breathing]). Each mouse was exposed to nebulized PBS (control) for 3 min, followed by nebulized Mch at increasing concentrations (6.25, 12.5, 25, and 50 mg/mL). Changes in airway resistance as the Mch concentration increased were recorded. An aerosonic ultrasonic nebulizer (DeVilbiss, Somerset, PA, USA) was used for nebulization.

### Bronchoalveolar lavage (BAL)

BAL was performed immediately after measurement of AHR. The trachea was exposed and cannulated with a silicone tube attached to a 23-gauge needle on a 1-mL tuberculin syringe. BAL fluid was withdrawn after administering 0.8 mL sterile PBS through the trachea. Total cell counts in BAL fluid were measured using a LUNA Automated Cell Counter (Logos Biosystems, Inc., Annandale, VA, USA). Each BAL fluid sample was cytospun at 2,000 rpm for 7 min, and the pellet was resuspended and placed on a microscope slide that was then stained with Diff-Quik (Sysmax, Kobe, Japan). The percentages of macrophages, neutrophils, and eosinophils were derived by counting 500 leukocytes of randomly selected fields under a light microscope. The supernatants were stored at –80ºC.

### Enzyme-linked immunosorbent assay (ELISA)

We used ELISA to measure the concentrations of interleukin (IL)-4, IL-5. and IL-13 in BAL fluid and IgE in serum using commercial kits, following the protocols of the manufacturer (R&D Systems). RELM-β levels were also measured by ELISA (LSBio, Seattle, WA, USA) using a standard protocol and a plate reader (PowerWave XS; BioTek, Winooski, VT, USA).

### Lung histopathology

After collecting BAL fluid, lungs were inflated and fixed in 4% (v/v) paraformaldehyde for 24 h and embedded in paraffin; 4-μm-thick sections were cut using a microtome, and the sections were deparaffinized and stained with hematoxylin and eosin (H&E) to assess airway inflammation. Inflammation scores were calculated based on inflammation distribution and severity, as described previously^51^: 0: no inflammation; 1: occasional inflammatory cells; 2: thin layer (1–5) of inflammatory cells surrounding most bronchi; 3: thick layer (> 5) of inflammatory cells surrounding most bronchi and vessels. Paraffin-embedded lung tissues were sectioned at a thickness of 5–6 μm and stained with periodic acid-Schiff (PAS) to detect goblet cell hyperplasia in the airway epithelium; this was graded from 0 to 4, as described previously^23^: 0: no goblet cells; 1: < 25% of cells; 2: 25–50% of cells; 3: 51–75% of cells; 4: > 75% of cells. The mean goblet cell hyperplasia score was calculated for each mouse.

### Measurement of smooth muscle area

Alpha-smooth muscle actin (α-SMA) was immunohistochemically detected as described previously^23^. Briefly, paraffin blocks were sectioned at a thickness of 6 μm, deparaffinized in xylene, rehydrated in ethanol, and incubated overnight at 4ºC with a primary monoclonal antibody against α-SMA (titer 1:50; Dako, High Wycombe, UK). Immunoreactivity was assessed by further incubation with a biotinylated secondary antibody, followed by peroxidase treatment (Vector Laboratories, Burlingame, CA, USA) and the addition of a diaminobenzidine chromogen (Invitrogen, Carlsbad, CA, USA). The α-SMA-immunostained area in each lung section was outlined and quantified using a light microscope and image analysis system (BX50; Olympus, Tokyo, Japan). The immunostained areas of the bronchiolar basement membranes (internal diameter = 150–200 μm) were derived. At least 10 bronchioles were counted in each slide.

### Hydroxyproline assay

Hydroxyproline assays were performed on 60 mg of the lung homogenate of each mouse using a colorimetric assay kit (BioVision, Milpitas, CA, USA) following the manufacturer’s instructions.

### Human blood collection and RELM-β measurement

We obtained blood samples from asthma patients (n = 44) and normal controls (n = 39) after approval was granted by the Institutional Review Board of the Catholic University of Korea Seoul St. Mary’s Hospital (approval no. KC15OIMI0553). Normal controls were those who visited medical center for general health assessment and had no respiratory symptoms or underlying respiratory diseases. Written informed consent was obtained from all subjects. Blood samples were collected and clotted, and serum was separated by centrifugation and stored at –80 °C. We measured RELM-β levels in the sera of asthmatic patients by ELISA (LSBio), using a standard protocol and a plate reader (PowerWave XS; BioTek).

### Statistical analysis

All data are presented as mean ± SEM or median [IQR] as appropriate. Results were compared among groups by analysis of variance (ANOVA) followed by the *post*-*hoc* Dunn multiple comparison test, and by t-test and the nonparametric Kruskal–Wallis test. GraphPad Prism (GraphPad Software Inc, San Diego, CA, USA) was used for all analyses. *P* < *0.05* was taken to indicate statistical significance.

## Supplementary Information

Supplementary Information 1.

Supplementary Information 2.

Supplementary Information 3.

## Data Availability

The datasets supporting the conclusions of this article are available from the corresponding author on reasonable request.

## References

[1] Olin JT, Wechsler ME (2014). Asthma: Pathogenesis and novel drugs for treatment. BMJ.

[2] Mattiuzzi C, Lippi G (2020). Worldwide asthma epidemiology: Insights from the Global Health Data Exchange database. Int. Forum Allergy Rhinol..

[3] GINA guideline, 2021. https://ginasthma.org/gina-reports/.

[4] Wenzel SE (2021). Severe adult asthmas: Integrating clinical features, biology, and therapeutics to improve outcomes. Am. J. Respir. Crit. Care Med..

[5] Hekking PW (2015). The prevalence of severe refractory asthma. J. Allergy Clin. Immunol..

[6] Wang E (2020). Characterization of severe asthma worldwide: Data from the international severe asthma registry. Chest.

[7] Sadatsafavi M (2010). Direct health care costs associated with asthma in British Columbia. Can. Respir. J..

[8] Fajt ML, Wenzel SE (2017). Development of new therapies for severe asthma. Allergy Asthma Immunol. Res..

[9] Rasmusson I (2006). Immune modulation by mesenchymal stem cells. Exp. Cell Res..

[10] Hipp J, Atala A (2008). Sources of stem cells for regenerative medicine. Stem. Cell Rev..

[11] Gao F (2016). Mesenchymal stem cells and immunomodulation: Current status and future prospects. Cell Death Dis..

[12] Habibian R, Delirezh N, Farshid AA (2018). The effects of bone marrow-derived mesenchymal stem cells on ovalbumin-induced allergic asthma and cytokine responses in mice. Iran. J. Basic Med. Sci..

[13] Kitoko JZ (2018). Therapeutic administration of bone marrow-derived mesenchymal stromal cells reduces airway inflammation without up-regulating Tregs in experimental asthma. Clin. Exp. Allergy.

[14] Dai R (2018). Intratracheal administration of adipose derived mesenchymal stem cells alleviates chronic asthma in a mouse model. BMC Pulm. Med..

[15] de Castro LL (2017). Human adipose tissue mesenchymal stromal cells and their extracellular vesicles act differentially on lung mechanics and inflammation in experimental allergic asthma. Stem Cell Res. Ther..

[16] Yu X, Yu L, Guo B, Chen R, Qiu C (2020). A narrative review of research advances in mesenchymal stem cell therapy for asthma. Ann. Transl. Med..

[17] Terraza-Aguirre C (2020). Mechanisms behind the immunoregulatory dialogue between mesenchymal stem cells and Th17 cells. Cells.

[18] Boldrini-Leite LM (2020). Lung tissue damage associated with allergic asthma in BALB/c mice could be controlled with a single injection of mesenchymal stem cells from human bone marrow up to 14 d after transplantation. Cell Transplant..

[19] Lambrecht BN, Hammad H (2015). The immunology of asthma. Nat. Immunol..

[20] Nemeth K (2010). Bone marrow stromal cells use TGF-beta to suppress allergic responses in a mouse model of ragweed-induced asthma. Proc. Natl. Acad. Sci. U.S.A..

[21] Aguiar FS (2020). Autologous bone marrow-derived mononuclear cell therapy in three patients with severe asthma. Stem Cell Res. Ther..

[22] Hur J (2020). Evaluation of human MSCs treatment frequency on airway inflammation in a mouse model of acute asthma. J. Korean Med. Sci..

[23] Choi JY (2018). TRPV1 blocking alleviates airway inflammation and remodeling in a chronic asthma murine model. Allergy Asthma Immunol. Res..

[24] Lin Q, Johns RA (2020). Resistin family proteins in pulmonary diseases. Am. J. Physiol. Lung Cell Mol. Physiol..

[25] WernstedtAsterholm I (2016). Pathological type-2 immune response, enhanced tumor growth, and glucose intolerance in Retnlβ (RELMβ) null mice: A model of intestinal immune system dysfunction in disease susceptibility. Am. J. Pathol..

[26] Pine GM, Batugedara HM, Nair MG (2018). Here, there and everywhere: Resistin-like molecules in infection, inflammation, and metabolic disorders. Cytokine.

[27] Fang CL (2015). Resistin-like molecule-β (RELM-β) targets airways fibroblasts to effect remodelling in asthma: From mouse to man. Clin. Exp. Allergy.

[28] Mishra A (2007). Resistin-like molecule-beta is an allergen-induced cytokine with inflammatory and remodeling activity in the murine lung. Am. J. Physiol. Lung Cell Mol. Physiol..

[29] LeMessurier KS, Palipane M, Tiwary M, Gavin B, Samarasinghe AE (2018). Chronic features of allergic asthma are enhanced in the absence of resistin-like molecule-beta. Sci. Rep..

[30] Kang HS (2016). Different anti-remodeling effect of nilotinib and fluticasone in a chronic asthma model. Korean J. Intern. Med..

[31] Rhee CK (2011). Effect of imatinib on airway smooth muscle thickening in a murine model of chronic asthma. Int. Arch. Allergy Immunol..

[32] Rhee CK (2014). Effect of nilotinib on airway remodeling in a murine model of chronic asthma. Exp. Lung Res..

[33] Kim SW (2016). Effect of roflumilast on airway remodelling in a murine model of chronic asthma. Clin. Exp. Allergy.

[34] Lee HY (2016). Effect of intranasal rosiglitazone on airway inflammation and remodeling in a murine model of chronic asthma. Korean J. Intern. Med..

[35] Lee HY (2017). Effect of nintedanib on airway inflammation and remodeling in a murine chronic asthma model. Exp. Lung Res..

[36] Firinci F (2011). Mesenchymal stem cells ameliorate the histopathological changes in a murine model of chronic asthma. Int. Immunopharmacol..

[37] Dalouchi F (2021). Human amniotic membrane mesenchymal stem cell-conditioned medium reduces inflammatory factors and fibrosis in ovalbumin-induced asthma in mice. Exp. Physiol..

[38] Ogulur I (2014). Suppressive effect of compact bone-derived mesenchymal stem cells on chronic airway remodeling in murine model of asthma. Int. Immunopharmacol..

[39] Royce SG, Mao W, Lim R, Kelly K, Samuel CS (2019). iPSC- and mesenchymoangioblast-derived mesenchymal stem cells provide greater protection against experimental chronic allergic airways disease compared with a clinically used corticosteroid. Faseb J..

[40] Royce SG, Rele S, Broughton BRS, Kelly K, Samuel CS (2017). Intranasal administration of mesenchymoangioblast-derived mesenchymal stem cells abrogates airway fibrosis and airway hyperresponsiveness associated with chronic allergic airways disease. Faseb J..

[41] Li Y (2017). Placenta-derived mesenchymal stem cells improve airway hyperresponsiveness and inflammation in asthmatic rats by modulating the Th17/Treg balance. Mol. Med. Rep..

[42] Kang SY (2017). Immunologic regulatory effects of human umbilical cord blood-derived mesenchymal stem cells in a murine ovalbumin asthma model. Clin. Exp. Allergy.

[43] Abreu SC (2017). Bone marrow, adipose, and lung tissue-derived murine mesenchymal stromal cells release different mediators and differentially affect airway and lung parenchyma in experimental asthma. Stem Cells Transl. Med..

[44] Fang SB (2018). Human iPSC-MSCs prevent steroid-resistant neutrophilic airway inflammation via modulating Th17 phenotypes. Stem Cell Res. Ther..

[45] Hong GH (2017). hMSCs suppress neutrophil-dominant airway inflammation in a murine model of asthma. Exp. Mol. Med..

[46] Lathrop MJ (2014). Mesenchymal stromal cells mediate Aspergillus hyphal extract-induced allergic airway inflammation by inhibition of the Th17 signaling pathway. Stem Cells Transl. Med..

[47] Lee SH (2011). Mesenchymal stem cell transfer suppresses airway remodeling in a toluene diisocyanate-induced murine asthma model. Allergy Asthma Immunol. Res..

[48] Martínez-González I (2014). Human mesenchymal stem cells resolve airway inflammation, hyperreactivity, and histopathology in a mouse model of occupational asthma. Stem Cells Dev..

[49] Fang C (2012). Resistin-like molecule-β is a human airway remodelling mediator. Eur. Respir. J..

[50] Yoon DS (2014). Interleukin-6 induces the lineage commitment of bone marrow-derived mesenchymal multipotent cells through down-regulation of Sox2 by osteogenic transcription factors. Faseb J..

[51] Li Z, Zheng J, Zhang N, Li C (2016). Berberine improves airway inflammation and inhibits NF-κB signaling pathway in an ovalbumin-induced rat model of asthma. J. Asthma.

