# Author Correction: Effects of human adipose tissue- and bone marrow-derived mesenchymal stem cells on airway inflammation and remodeling in a murine model of chronic asthma

**DOI:** 10.1038/s41598-022-24092-x

**Published:** 2022-11-16

**Authors:** Joon Young Choi, Jung Hur, Sora Jeon, Chan Kwon Jung, Chin Kook Rhee

**Affiliations:** 1 Division of Pulmonary and Critical Care Medicine, Department of Internal Medicine, Incheon St. Mary’s Hospital, College of Medicine, The Catholic University of Korea, Seoul, Republic of Korea; 2 Division of Pulmonary and Critical Care Medicine, Department of Internal Medicine, Seoul St. Mary’s Hospital, College of Medicine, The Catholic University of Korea, 222 Banpodaero, Seochogu, Seoul, 06591 Republic of Korea; 3 Cancer Research Institute, College of Medicine, The Catholic University of Korea, Seoul, Republic of Korea; 4 Department of Hospital Pathology, Seoul St. Mary’s Hospital, College of Medicine, The Catholic University of Korea, 222 Banpodaero, Seochogu, Seoul, 06591 Republic of Korea

Correction to: *Scientific Reports* 10.1038/s41598-022-16165-8 published online 14 July 2022

The original version of the Article contained errors. The spelling of the author name Chan Kwon Jung was incorrectly given as Chang Kwon Jung.

In addition, the Author Information section was incorrect.

“These authors contributed equally: Joon Young Choi, Jung Hur, Chang Kwon Jung and Chin Kook Rhee.”

now reads:

“These authors contributed equally: Joon Young Choi and Jung Hur.

These authors jointly supervised this work: Chan Kwon Jung and Chin Kook Rhee.”

The original Article has been corrected.

