# Supplementary material for: Effects of human adipose tissue- and bone marrow-derived mesenchymal stem cells on airway inflammation and remodeling in a murine model of chronic asthma
Source: Sci Rep. 2022 Jul 14;12:12032. doi: 10.1038/s41598-022-16165-8 (PMC9283392; doi:10.1038/s41598-022-16165-8)
Supplement: Supplementary file 1 — Supplementary Information 1. [file 41598_2022_16165_MOESM1_ESM.pdf]

세포 분양 목록

code no.	code	Sex	Age	혈액형	채취부위	BMI	진단명	수술명
6	A100112	F	55	A+	Breast	26.56	Macromastia	Reduction mammplasty
7	A100216	F	30	O+	abdomen	40.75	skin laxity, abdomen and back	Dermolipectomy and abdominoplasty
17	A110929	F	54	O+	Abdomen	20.35	Breast ca., Lt.	TRAM
21	A111018	F	41	A+	Abdomen	27.83	Breast ca., Rt.	DIEP coverage
23	A1111031	F	39	O+	Abdomen	22.01	Breast ca., Lt.	DIEP coverage
25	A111116	M	49	A+	Abdomen	31.71	Skin laxity, abdomen	Abdominoplasty c liposuction
26	A111117	F	64	O+	Abdomen	23.53	Breast ca., Lt.	TRAM flap coverage
27	A111129	F	43	A+	Abdomen	27.43	Obesity, abdomen, back & thigh	Liposuction abdominoplasty c SAL
28	A111215	F	40	AB+	Abdomen	21.21	Breast ca., Lt.	DIEP coverage
29	A120507	M	18	A+	Abdomen	22.45	Short nose<br>Scar, upper eyelid, Rt.<br>Depression, lower eyelid, Rt.	Fat injection
30	A111226	F	22	A+	Buttock	20.83	iant hairy nevus, buttock, Lt	2nd staged excision
32	A120208	M	72	A+	Neck	19.14	Lipomatosis, post. Neck	Excision and local flap coverage
42	A120227	F	52	A+	Breast	22.66	Breast ca., Lt. (s/p MRM)	Delayed breast reconstruction with contralateral free DIEP flap coverage
46	A120315	F	49	B+	Abdomen	23.06	Breast ca., Lt.	Immediate breast recon c ipsilat. free DIEP flap coverage
47	A120321	F	41	O+	Abdomen	27.14	Fatty abdomen, upper arm & thigh, both	Abdominoplasty, Suction assisted lipectomy
56	A120403	F	45	B+	Abdomen	29.55	Fatty abdomen/Accessory breast, Lt./Dog ear, breast, both	Lipobdominoplasty/Ex. & Bx./ Revision
71	A161205	F	47		skin	20.45	flame burn	

분양 세포 정보

code no.	code	Sex	Age	혈액형	채취부위	BMI	진단명	수술명
6	A100112	F	55	A+	Breast	26.56	Macromastia	Reduction mammplasty

Cell info.

code no.	code	세포 배가 시간	세포 생존율	콜로니 형성수	동결 cell viability (%)	분화능	FACS
6	A100112	O	O	O	O	O	O

세포 배가 시간	세포 생존율	콜로니 형성수	동결 cell viability (%)	분화능	FACS
**평균**	**평균**	**평균**	**Cell stock 후 6개월**	**control**	**CD13**
48.0	86.21	101	90.00%	0.044	98.4
			**Cell stock 후 12개월**	**osteo**	**CD90**
			90.94%	0.234	86.9
			**Cell stock 후 18개월**	**intensity**	**CD73**
			90.23%	5.368	81.5
				**control**	**CD105**
				0.852	82.5
				**adipo**	**CD34**
				1.239	1.1
				**intensity**	**CD45**
				1.454	0
					**CD146**
					4.4
					**HLA**
					17.5

**Positive**
**Negative**

code no.	code	Sex	Age	혈액형	채취부위	BMI	진단명	수술명
7	A100216	F	30	O+	abdomen	40.75	skin laxity, abdomen and back	Dermolipectomy and abdominoplasty

Cell info.

code no.	code	세포 배가 시간	세포 생존율	콜로니 형성수	동결 cell viability (%)	분화능	FACS
7	A100216	O	O	O	O	O	O

세포 배가 시간	세포 생존율	콜로니 형성수	동결 cell viability (%)	분화능	FACS
**평균**	**평균**	**평균**	**Cell stock 후 6개월**	**control**	**CD13**
33.6	88.96	40	81.97%		99.2
			**Cell stock 후 12개월**	**osteo**	**CD90**
			91.24%		98.3
			**Cell stock 후 18개월**	**intensity**	**CD73**
			92.89%		97
				**control**	**CD105**
					97
				**adipo**	**CD34**
					1
				**intensity**	**CD45**
					0
					**CD146**
					6.2
					**HLA**
					0.3

**Positive**
**Negative**

code no.	code	Sex	Age	혈액형	채취부위	BMI	진단명	수술명
17	A110929	F	54	O+	Abdomen	20.35	Breast ca., Lt.	TRAM

Cell info.

code no.	code	세포 배가 시간	세포 생존율	콜로니 형성수	동결 cell viability (%)	분화능	FACS
17	A110929	O	O	O	O	O	O
		평균	평균	평균	Cell stock 후 6개월	control	CD13
		52.1	96.14	160	93.87%	0.055	97.8
					Cell stock 후 12개월	osteo	CD90
					88.86%	0.275	99.6
					Cell stock 후 18개월	intensity	CD73
					93.59%	5.000	87
						control	CD105
						0.695	97.9
						adipo	CD34
						1.090	1.9
						intensity	CD45
						1.568	0
							CD146
							3.4
							HLA
							3.7

Positive
Negative

code no.	code	Sex	Age	혈액형	채취부위	BMI	진단명	수술명
21	A111018	F	41	A+	Abdomen	27.83	Breast ca., Rt.	DIEP coverage

Cell info.

code no.	code	세포 배가 시간	세포 생존율	콜로니 형성수	동결 cell viability (%)	분화능	FACS
21	A111018	O	O	O	O	O	O
		평균	평균	평균	Cell stock 후 6개월	control	CD13
		65.6	94.32	117	94.77%	0.085	99.3
					Cell stock 후 12개월	osteo	CD90
					91.79%	0.423	98.5
					Cell stock 후 18개월	intensity	CD73
					91.94%	4.976	95.3
						control	CD105
						0.915	95.8
						adipo	CD34
						1.410	0.3
						intensity	CD45
						1.541	0
							CD146
							8.2
							HLA
							0.2

Positive
Negative

code no.	code	Sex	Age	혈액형	채취부위	BMI	진단명	수술명
23	A1111031	F	39	O+	Abdomen	22.01	Breast ca., Lt.	DIEP coverage

Cell info.

code no.	code	세포 배가 시간	세포 생존율	콜로니 형성수	동결 cell viability (%)	분화능	FACS
23	A1111031	O	O	O	O	O	O
		평균	평균	평균	Cell stock 후 6개월	control	CD13
		56.3	89.20	133	93.10%	0.075	99.4
					Cell stock 후 12개월	osteo	CD90
					90.46%	0.372	99.8
					Cell stock 후 18개월	intensity	CD73
					93.89%	4.960	92.1
						control	CD105
						0.825	95.1
						adipo	CD34
						1.210	1
						intensity	CD45
						1.467	0
							CD146
							2
							HLA
							0.1

Positive
Negative

code no.	code	Sex	Age	혈액형	채취부위	BMI	진단명	수술명
25	A111116	M	49	A+	Abdomen	31.71	Skin laxity, abdomen	Abdominoplasty c liposuction

Cell info.

code no.	code	세포 배가 시간	세포 생존율	콜로니 형성수	동결 cell viability (%)	분화능	FACS
25	A111116	O	O	O	O	O	O

평균	평균	평균	Cell stock 후 6개월	control	CD13
84.4	86.13	63	88.87%	0.065	99.9
			Cell stock 후 12개월	osteo	CD90
			88.89%	0.371	94.6
			Cell stock 후 18개월	intensity	CD73
			93.62%	5.708	85.7
				control	CD105
				0.795	80.5
				adipo	CD34
				1.560	0.8
				intensity	CD45
				1.962	0.3
					CD146
					2.9
					HLA
					0.9

Positive
Negative

code no.	code	Sex	Age	혈액형	채취부위	BMI	진단명	수술명
26	A111117	F	64	O+	Abdomen	23.53	Breast ca., Lt.	TRAM flap coverage

Cell info.

code no.	code	세포 배가 시간	세포 생존율	콜로니 형성수	동결 cell viability (%)	분화능	FACS
26	A111117	O	O	O	O	O	O

평균	평균	평균	Cell stock 후 6개월	control	CD13
90.2	91.52	119	94.89%	0.075	99.8
			Cell stock 후 12개월	osteo	CD90
			92.50%	0.359	95.2
			Cell stock 후 18개월	intensity	CD73
			94.66%	4.787	84
				control	CD105
				0.895	86.4
				adipo	CD34
				1.230	2.4
				intensity	CD45
				1.374	0.9
					CD146
					3.1
					HLA
					1

Positive
Negative

code no.	code	Sex	Age	혈액형	채취부위	BMI	진단명	수술명
27	A111129	F	43	A+	Abdomen	27.43	Obesity, abdomen, back & thigh	Liposuction abdominoplasty c SAL

Cell info.

code no.	code	세포 배가 시간	세포 생존율	콜로니 형성수	동결 cell viability (%)	분화능	FACS
27	A111129	O	O	O	O	O	O

평균	평균	평균	Cell stock 후 6개월	control	CD13
174.4	94.45	88	89.52%	0.051	96.2
			Cell stock 후 12개월	osteo	CD90
			91.77%	0.271	95.8
			Cell stock 후 18개월	intensity	CD73
			95.65%	5.314	99
				control	CD105
				1.051	70.4
				adipo	CD34
				1.548	0.4
				intensity	CD45
				1.473	0.1
					CD146
					2.8
					HLA
					0.1

Positive
Negative

code no.	code	Sex	Age	혈액형	채취부위	BMI	진단명	수술명
28	A111215	F	40	AB+	Abdomen	21.21	Breast ca., Lt.	DIEP coverage

Cell info.

code no.	code	세포 배가 시간	세포 생존율	콜로니 형성수	동결 cell viability (%)	분화능	FACS
28	A111215	O	O	O	O	O	O

평균	평균	평균	Cell stock 후 6개월	control	CD13
56.0	95.27	125	81.53%	0.057	93.6
			Cell stock 후 12개월	osteo	CD90
			92.71%	0.254	99.4
			Cell stock 후 18개월	intensity	CD73
			92.68%	4.982	92.8
				control	CD105
				0.697	97.7
				adipo	CD34
				1.098	5.5
				intensity	CD45
				1.575	0.1
					CD146
					1.1
					HLA
					0.2

Positive
Negative

code no.	code	Sex	Age	혈액형	채취부위	BMI	진단명	수술명
29	A120507	M	18	A+	Abdomen	22.45	Short nose<br>Scar, upper eyelid, Rt.<br>Depression, lower eyelid, Rt.	Fat injection

Cell info.

code no.	code	세포 배가 시간	세포 생존율	콜로니 형성수	동결 cell viability (%)	분화능	FACS
29	A120507	O	O	O	O	O	O

평균	평균	평균	Cell stock 후 6개월	control	CD13
61.7	98.65	84	89.75%	0.070	97.7
			Cell stock 후 12개월	osteo	CD90
			91.70%	0.327	99.4
			Cell stock 후 18개월	intensity	CD73
			93.18%	4.671	96.3
				control	CD105
				0.830	81.4
				adipo	CD34
				1.320	2.5
				intensity	CD45
				1.590	0
					CD146
					0.2
					HLA
					0.1

Positive
Negative

code no.	code	Sex	Age	혈액형	채취부위	BMI	진단명	수술명
30	A111226	F	22	A+	Buttock	20.83	iant hairy nevus, buttock, Lt	2nd staged excision

Cell info.

code no.	code	세포 배가 시간	세포 생존율	콜로니 형성수	동결 cell viability (%)	분화능	FACS
30	A111226	O	O	O	O	O	O

평균	평균	평균	Cell stock 후 6개월	control	CD13
39.8	96.62	101	92.51%	0.069	98.4
			Cell stock 후 12개월	osteo	CD90
			94.52%	0.295	99.3
			Cell stock 후 18개월	intensity	CD73
			93.09%	4.275	95.4
				control	CD105
				0.719	72.1
				adipo	CD34
				1.050	5.3
				intensity	CD45
				1.460	0.1
					CD146
					0.5
					HLA
					0.1

Positive
Negative

code no.	code	Sex	Age	혈액형	채취부위	BMI	진단명	수술명
32	A120208	M	72	A+	Neck	19.14	Lipomatosis, post. Neck	Excision and local flap coverage

Cell info.

code no.	code	세포 배가 시간	세포 생존율	콜로니 형성수	동결 cell viability (%)	분화능	FACS
32	A120208	O	O	O	O	O	O

평균	평균	평균	Cell stock 후 6개월	control	CD13
38.0	92.11	75	91.13%	0.091	98.3
			Cell stock 후 12개월	osteo	CD90
			92.57%	0.407	99.8
			Cell stock 후 18개월	intensity	CD73
			95.64%	4.477	94.4
				control	CD105
				0.951	55
				adipo	CD34
				1.261	0.6
				intensity	CD45
				1.326	0.2
					CD146
					1.2
					HLA
					0.3

Positive
Negative

code no.	code	Sex	Age	혈액형	채취부위	BMI	진단명	수술명
42	A120227	F	52	A+	Breast	22.66	Breast ca., Lt. (s/p MRM)	Delayed breast reconstruction with contralateral free DIEP flap coverage

Cell info.

code no.	code	세포 배가 시간	세포 생존율	콜로니 형성수	동결 cell viability (%)	분화능	FACS
42	A120227	O	O	O	O	O	O

평균	평균	평균	Cell stock 후 6개월	control	CD13
27.4	91.97	56	87.80%	0.067	97.6
			Cell stock 후 12개월	osteo	CD90
			89.09%	0.293	98.5
			Cell stock 후 18개월	intensity	CD73
			94.18%	4.373	95.3
				control	CD105
				0.676	75.2
				adipo	CD34
				0.816	1.8
				intensity	CD45
				1.207	0.1
					CD146
					3.9
					HLA
					0.1

Positive
Negative

code no.	code	Sex	Age	혈액형	채취부위	BMI	진단명	수술명
46	A120315	F	49	B+	Abdomen	23.06	Breast ca., Lt.	Immediate breast recon c ipsilat. free DIEP flap coverage

Cell info.

code no.	code	세포 배가 시간	세포 생존율	콜로니 형성수	동결 cell viability (%)	분화능	FACS
46	A120315	O	O	O	O	O	O

평균	평균	평균	Cell stock 후 6개월	control	CD13
28.9	93.38	72	94.03%	0.074	96.3
			Cell stock 후 12개월	osteo	CD90
			92.44%	0.314	98.4
			Cell stock 후 18개월	intensity	CD73
			93.00%	4.243	92.2
				control	CD105
				0.644	85.4
				adipo	CD34
				0.724	0.6
				intensity	CD45
				1.124	0.1
					CD146
					1.1
					HLA
					0.1

Positive
Negative

code no.	code	Sex	Age	혈액형	채취부위	BMI	진단명	수술명
47	A120321	F	41	O+	Abdomen	27.14	Fatty abdomen, upper arm & thigh, both	Abdominoplasty, Suction assisted lipectomy

Cell info.

code no.	code	세포 배가 시간	세포 생존율	콜로니 형성수	동결 cell viability (%)	분화능	FACS
47	A120321	O	O	O	O	O	O

평균	평균	평균	Cell stock 후 6개월	control	CD13
65.7	97.22	80	88.33%	0.076	99.9
			Cell stock 후 12개월	osteo	CD90
			93.50%	0.349	90.6
			Cell stock 후 18개월	intensity	CD73
			91.68%	4.592	86.8
				control	CD105
				0.534	81.8
				adipo	CD34
				0.821	0.8
				intensity	CD45
				1.537	0.6
					CD146
					1.4
					HLA
					0.8

Positive
Negative

code no.	code	Sex	Age	혈액형	채취부위	BMI	진단명	수술명
56	A120403	F	45	B+	Abdomen	29.55	Fatty abdomen/Accessory breast, Lt./Dog ear, breast, both	Lipobdominoplasty/Ex. & Bx./ Revision

Cell info.

code no.	code	세포 배가 시간	세포 생존율	콜로니 형성수	동결 cell viability (%)	분화능	FACS
56	A120403	O	O	O	O	O	O

평균	평균	평균	Cell stock 후 6개월	control	CD13
108.7	84.19	116	85.63%	0.077	100
			Cell stock 후 12개월	osteo	CD90
			90.17%	0.351	98.1
			Cell stock 후 18개월	intensity	CD73
			89.81%	4.558	89.4
				control	CD105
				0.314	81.7
				adipo	CD34
				0.339	4.1
				intensity	CD45
				1.080	0.6
					CD146
					0.7
					HLA
					1.6

Positive
Negative

code no.	code	Sex	Age	혈액형	채취부위	BMI	진단명	수술명
71	A161205	F	47		skin	20.45	flame burn	

Cell info.

code no.	code	세포 배가 시간	세포 생존율	콜로니 형성수	동결 cell viability (%)	분화능	FACS
71	A161205	O	O	O	O	O	O

d

평균	평균	평균	Cell stock 후 6개월	control	CD13
29.7	99.27	93		0.047	99.6
			Cell stock 후 12개월	osteo	CD90
				0.274	93.7
			Cell stock 후 18개월	intensity	CD73
				5.869	98.2
				control	CD105
				0.039	95.2
				adipo	CD34
				0.663	0.2
				intensity	CD45
				16.864	0.4
					CD146
					0
					HLA
					0.2

Positive
Negative
