# Supplementary figures and images for: Effects of human adipose tissue- and bone marrow-derived mesenchymal stem cells on airway inflammation and remodeling in a murine model of chronic asthma
Source: Sci Rep. 2022 Jul 14;12:12032. doi: 10.1038/s41598-022-16165-8 (PMC9283392; doi:10.1038/s41598-022-16165-8)

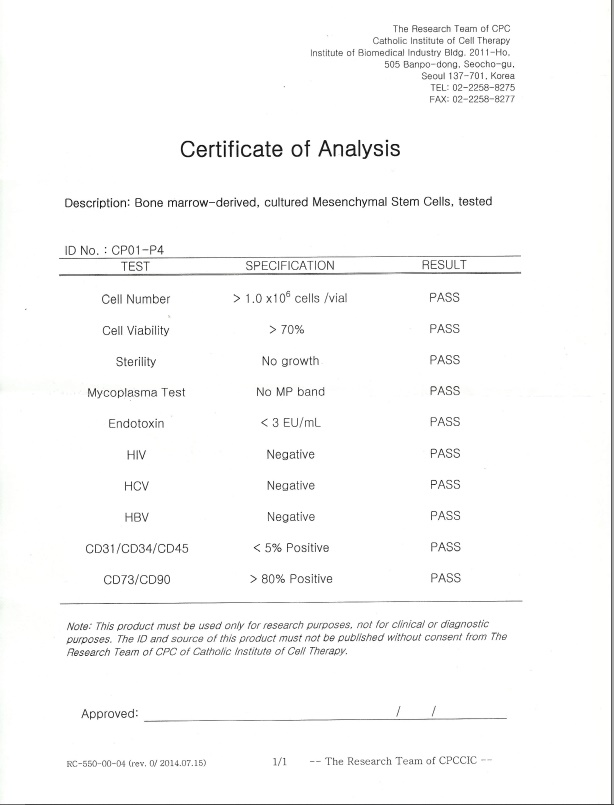

Supplement: Supplementary file 2 — Supplementary Information 2. [file 41598_2022_16165_MOESM2_ESM.jpg]

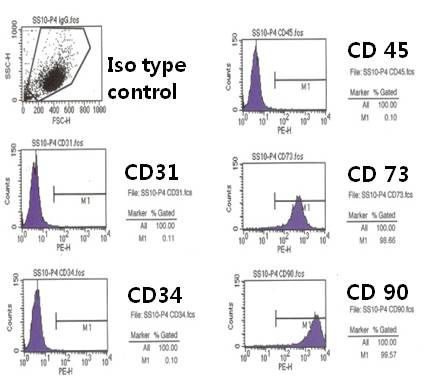

Supplement: Supplementary file 3 — Supplementary Information 3. [file 41598_2022_16165_MOESM3_ESM.jpg]
